# Acute fatal posthypoxic leukoencephalopathy following benzodiazepine overdose: a case report and review of the literature

**DOI:** 10.1186/s12883-015-0320-6

**Published:** 2015-04-30

**Authors:** Salman Aljarallah, Fawaz Al-Hussain

**Affiliations:** Department of Medicine, King Khalid University Hospital, College of Medicine, King Saud University, Riyadh, Saudi Arabia

**Keywords:** Delayed post-hypoxic leukoencephalopathy, Leukoencephalopathy, Benzodiazepines, Alprazolam, Coma, Hypoxia, Herniation, Demyelination, Acidosis, Toxin, Drugs of abuse

## Abstract

**Background:**

Among the rare neurological complications of substances of abuse is the selective cerebral white matter injury (leukoencephalopathy). Of which, the syndrome of delayed post hypoxic encephalopathy (DPHL) that follows an acute drug overdose, in addition to “chasing the dragon” toxicity which results from chronic heroin vapor inhalation remain the most commonly described syndromes of toxic leukoencephalopathy. These syndromes are reported in association with opioid use. There are very few cases in the literature that described leukoencephalopathy following benzodiazepines, especially with an acute and progressive course. In this paper, we present a patient who developed an acute severe fatal leukoencephalopathy following hypoxic coma and systemic shock induced by benzodiazepine overdose.

**Case presentation:**

A 19-year-old male was found comatose at home and brought to hospital in a deep coma, shock, hypoxia, and acidosis. Brain magnetic resonant imaging (MRI) revealed a strikingly selective white matter injury early in the course of the disease. The patient remained in a comatose state with no signs of neurologic recovery until he died few weeks later following an increase in the brain edema and herniation.

**Conclusion:**

Toxic leukoencephalopathy can occur acutely following an overdose of benzodiazepine and respiratory failure. This is unlike the usual cases of toxic leukoencephalopathy where there is a period of lucidity between the overdose and the development of white matter disease. Unfortunately, this syndrome remains of an unclear pathophysiology and with no successful treatment.

## Background

Leukoencephalopathies constitutes a group of neurological disorders characterized by diffuse damage to the cerebral white matter with relative sparing of the gray matter. In addition to the known genetic and metabolic syndromes, this entity has been associated with the use of different chemicals; the list includes therapeutic medications e.g. chemotherapy agents [1], toxins as in the case of carbon monoxide poisoning [2] or recreational drugs. Opioids are known to cause a picture of leukoencephalopathy since 1970s [3]. They are known to cause two distinct clinical syndromes depending on the context. The first is subacute/chronic neurologic decline with leukoencephalopathy that is associated with the chronic use of heroin vapor or the so called chasing the dragon toxicity (as this specific type of opioid preparation is known among the users as “chasing the dragon”) [4]. The other syndrome is less common and –typically- manifests itself as delayed manifestations few weeks following a near-complete recovery from a reversible hypoxic coma caused by opioid overdose. The latter condition is known in the literature as delayed post-hypoxic leukoencephalopathy (DPHL) [5-10]. It was also seen following other causes of coma like carbon monoxide poisoning [11] or cocaine [4].

Here, we present a patient who developed an unusually acute fatal leukoencephalopathy after an episode of hypoxic coma induced by an overdose of alprazolam.

At the time of the preparation of this manuscript, we were aware of only two publications of leukoencephalopathy associated with the sole use of benzodiazepines [8,12].

## Case presentation

A 19-year-old male was brought to the emergency room after being found unconscious at home. His father found him unresponsive with irregular noisy breathing and frothy salivation. An empty pack of Alprazolam (Xanax) was noted beside his bed. His father did not notice any jerky movements or classic seizure manifestation. The family denied any history of trauma, headache or seizures. Apart from easy irritability, tendency to spend most the time alone and sleep difficulties, the patient did not have major psychiatric disorder. He was a cigarette smoker and might have used illicit drugs. His past medical history was unremarkable apart from bronchial asthma exacerbations that necessitated admissions during his early childhood with no recurrence in adulthood.

Upon arrival, he was in deep coma (Glasgow Coma Scale score of 3 out of 15) with bilateral pinpoint unreactive pupils. There was no spontaneous voluntary or involuntary movement noted. He was hypotensive (systolic blood pressure of 70–80 mmHg), tachycardiac (heart rate of 132 beats/minute) and tachypniec (respiratory rate of 23 breaths/minute). His core body temperature was 38.8°C and oxygen saturation by pulse oximetry was 71%. Exam showed generalized hypotonia, diminished reflexes, absent plantar response with no meningeal signs. He had one episode of coffee-ground vomiting.

He was intubated, mechanically ventilated and resuscitated with intravenous fluids, vasopressors and inotropes (norepinephrine and dopamine). Arterial blood gases showed a picture of respiratory and metabolic acidosis (pH: 7.1, pCO2: 64.2 mmHg, PO2 57.8 mmHg and HCO3 20.9 mEq/L). His complete blood count showed leukocytosis (white blood cells count of 27.5 × 10e9/L, (80% neutrophils), a hemoglobin of 14.8 mg/dl, and platelet count of 214 × 10e9/L. He had acute kidney injury (creatinine 206 umol/L) with hyperkalemia (5.7 mmol/L). His liver function test showed highly elevated transaminases (aspartate aminotransferase of 326 U/L, alanine aminotransferase of 326 U/L) with normal alkaline phosphatase (ALP), gamma-glutamyltransferase (GGT), bilirubin and albumin. Coagulation profile showed INR of 2 with prolongation of PT and APTT. His serum creatine phosphokinase (CPK) level was 4858 U/L.

**Figure 1:**
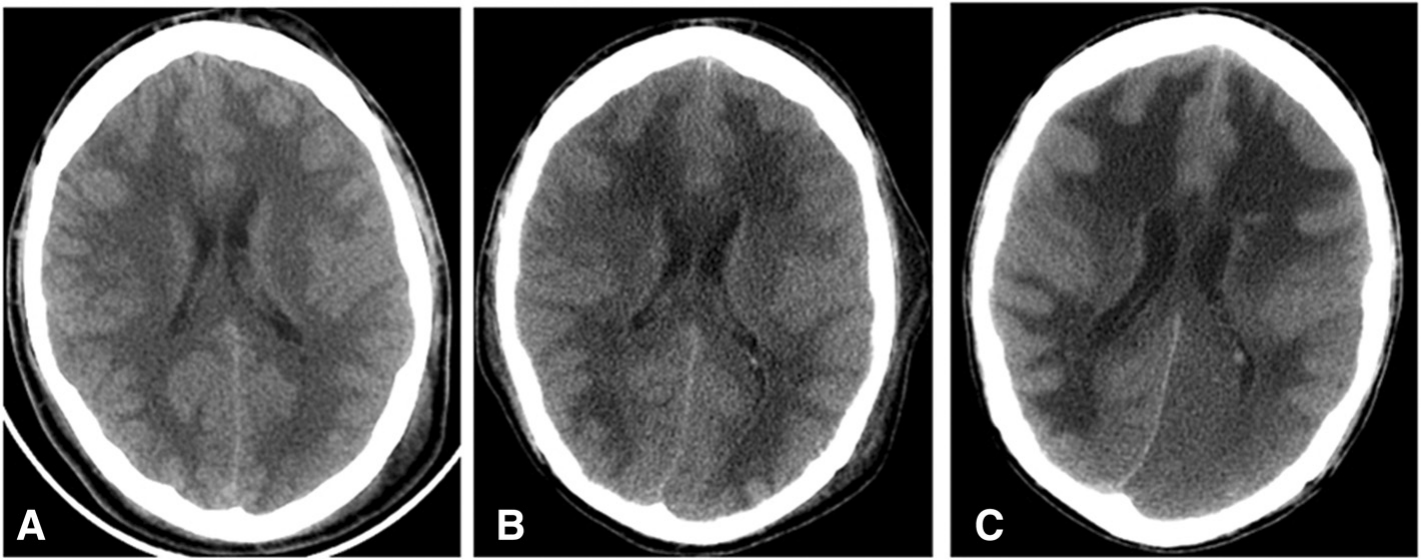
Brain CT scans throughout the hospital stay. **A)** admission CT scan showing an unremarkable brain parenchyma with preserved grey-white matter demarcation. There was also small left frontal scalp hematoma (not shown). **B)** A follow-up CT showing decreasing attenuation of the white matter diffusely. **C)** CT done 3 weeks of admission following patient clinical deterioration revealing evidence of progressive diffuse hypoattenuation the cerebral white matter, basal ganglia and thalami bilaterally. There is left occipital gray-white matter hypodensity consistent with infarct, explained by trantentorial herniation trapping the left posterior cerebral artery against tentorium cerebelli.

A chest X-ray done in the day of presentation showed an evidence of bilateral lung infiltrates more in the left side. A computed tomography (CT) scan of the brain without contrast done in the same day did not reveal any hemorrhage, space occupying lesion or territorial infarction. The brain parenchyma looked normal with preserved grey-white matter differentiation (Figure 1A).

His urine tested highly positive for benzodiazepines (>1000 ng/mL) and tetrahydrocannabinol (THC >100 ng/mL) while it did not show any traces of opioids, ethanol, amphetamines or cocaine. His plasma benzodiazepine level was 56.5 ng/mL. The patient was transferred to the ICU with the diagnoses of drug overdose, acute kidney injury, acute liver injury (toxic versus ischemic), aspiration pneumonia with respiratory failure and septic shock. Supportive measures were continued and antibiotics, intravenous proton pump inhibitors, vitamin K, fresh frozen plasma were given.

The next day, the patient improved in terms of hemodynamics and respiratory condition. His level of consciousness slightly improved as he started to open his eyes spontaneously, blinked to visual threat, extended his arms to painful stimulus but was not following commands and there was no verbal output. His blood leucocyte count started to subside gradually, and his liver function tests, urea and creatinine returned to near-baseline within days. All of his blood and sputum cultures did not grow any organisms. Human immunodeficiency virus (HIV) antibodies were negative by Enzyme-linked immunosorbent assay (ELISA) and the screening for hepatitis B and C was also negative. He had an episode of generalized tonic-clonic convulsion and levetiracetam was started via nasogastric tube.

Electroencephalography (EEG) was done at the same day of the seizure which revealed a diffuse severe slowing of background at 2–3 Hz of low voltage. However, there was no focality, asymmetry or epileptic abnormalities. Transthoracic echocardiography was done which showed an evidence of biventricular moderate to severe global systolic dysfunction.

Clinically, there was no further improvement noted in the following three days.

**Figure 2:**
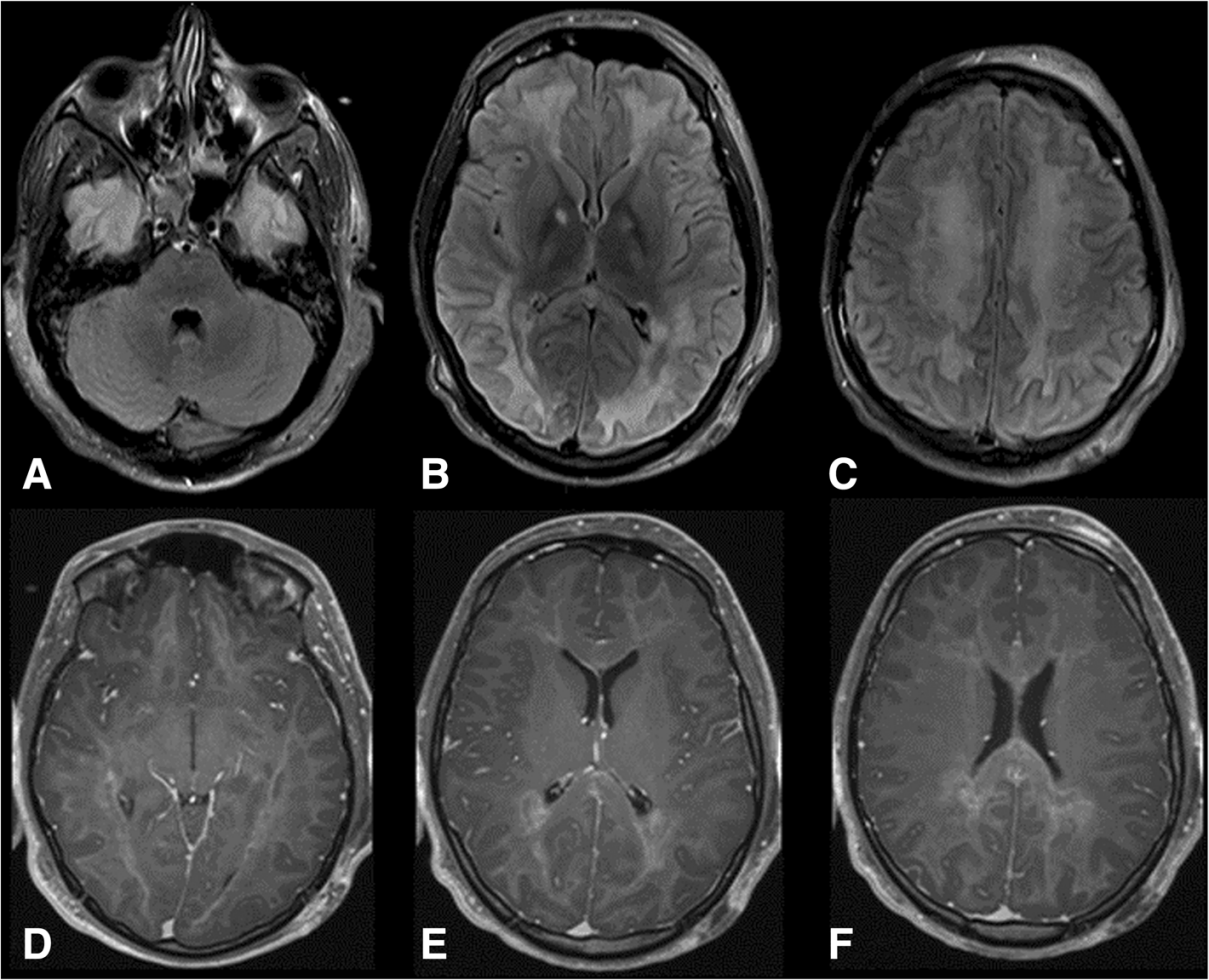
Magnetic resonant images of the brain. **A-C)** FLAIR images shows diffusely increased signal intensity in cerebral white matter. Small hyperintense foci are seen in globus pallidus bilaterally which is likely ischemic secondary to hypoxia. Left frontal scalp hematoma is seen. **D-F)** Post contrast T1 weighted images show minimal patchy enhancement is seen in cerebral white matter.

**Figure 3:**
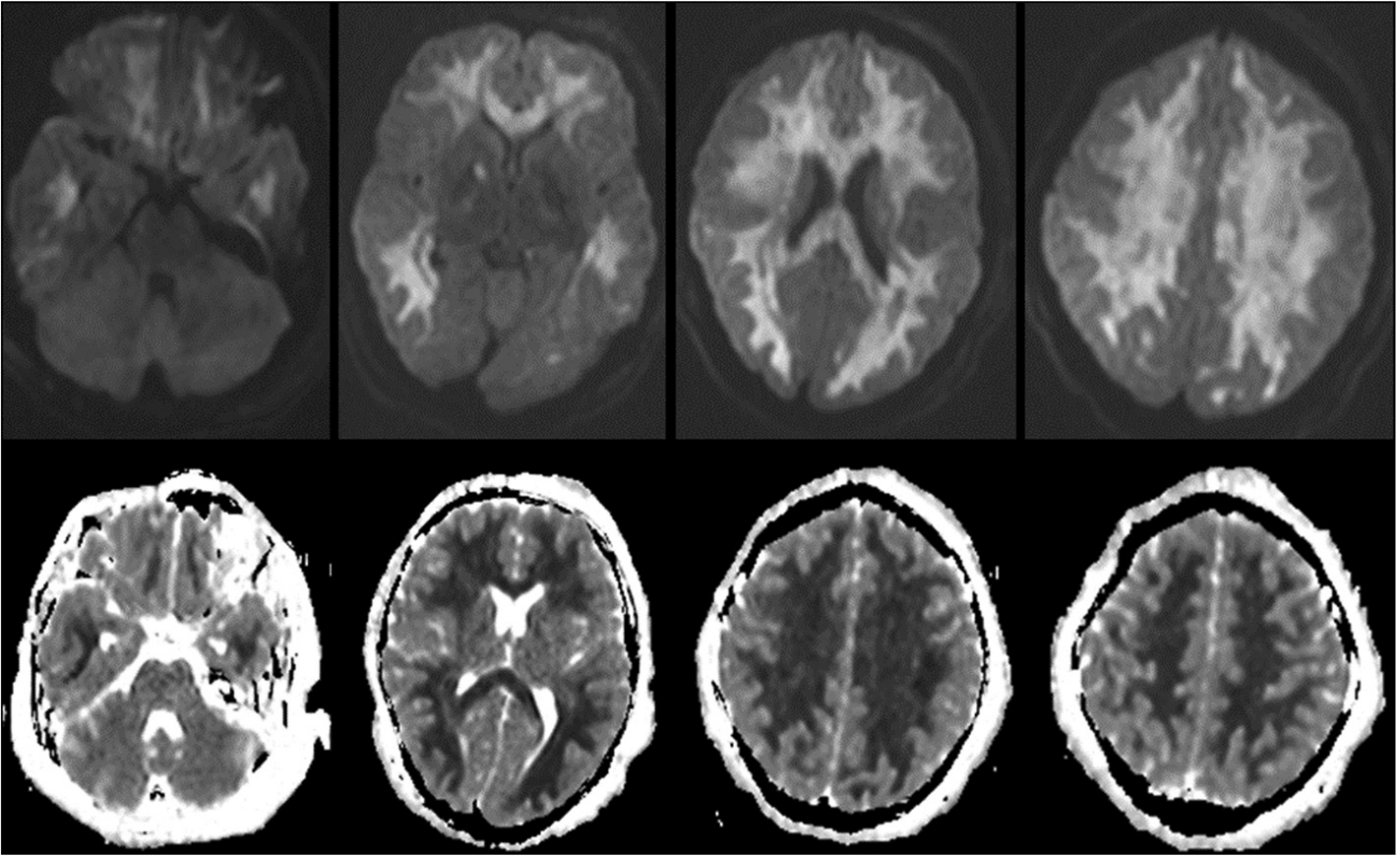
Diffusion weighted images (DWI). DWI in the upper row and apparent diffusion coefficient (ADC) maps in the lower row show areas of diffuse and symmetric diffusion restriction in the cerebral subcortical white matter and right globus pallidus that is corresponding to the changes seen in FLAIR images. Note the sparing of infratentorial structures.

In the fifth day of admission, he was found to have fixed dilated left pupil and normal reactive right one. His level of consciousness was the same. Physical examination showed generalized hypotonia with bilateral extensor plantar responses. Intravenous mannitol was given and urgent CT scan of the brain showed a clear generalized diffuse hypodensity involving the subcortical white matter (Figure 1B). MRI brain done in the 7th day of admission which showed diffuse white matter changes with sparing of the brain stem and cerebellum (Figures 2 and 3).

The patient spent 3 weeks intubated in the intensive care unit with full supportive care while his level of consciousness stayed at Glasgow come scale of 7 (opening eyes spontaneously and extending limbs to pain).

In the 23rd day of admission, the patient had bilaterally dilated unreactive pupils with absence of brainstem reflexes. CT scan of the brain showed increasing in the white matter hypodensity with evidence of tonsillar herniation and subsequent new left occipital infarction in the posterior cerebral artery territory. The patient was declared brain dead after fulfilling the brain death criteria.

## Conclusions

The MRI findings in our case shows extensive selective white matter damage with sparing of the gray matter. Strikingly, the brainstem and cerebellum were totally spared. Such findings resemble the cases of delayed post hypoxic leukoencephalopathy related to carbon monoxide or opioids overdose. The findings were more pronounced in the diffusion MRI sequences which suggest an acuteness of the illness.

The syndrome of post-hypoxic leukoencephalopathy occurs as a consequence of hypoxia that is brought by different causes. Most commonly, hypoxia induced by carbon monoxide poisoning [2] or opioid overdose, whither it is a recreational intravenous heroin or medically-used opioids for anesthesia or oral analgesia [5,9,10,13,14]. However, it was also described in association with cocaine [15] and benzodiazepine overdose [4,8]. To a lesser extent, this syndrome of post hypoxic leukoencephalopathy was also reported following cardiorespiratory arrest, shock [3,15-17] and metabolic coma e.g. hepatic, hyper or hypoglycemic [15]. In the next text, we will review the different presentation of toxic related hypoxic leukoencephalopathy.

After reviewing the literature, two types of post hypoxic leukoencephalopathy can be distinguished in relation to onset of symptoms after hypoxia. The more common is the delayed type (delayed post hypoxic leukoencephalopathy, DPHL), where the patient recovers to baseline after the coma and respiratory arrest with normal brain imaging initially (if done) and usually is sent home [5,6,9,16]. Afterwards, following a lucid period (>12 days) of return to normal function, the clinical manifestations appears with radiological evidence of diffuse leukoencephalopathy. The other less common type is the early or acute type where the patient's brain imaging shows an evidence of leukoencephalopathy in the first few days after the initial event without notable period of recovery [3,4,15,18]. In the next paragraphs, our main focus will be on the delayed type as it is more commonly described in the literature and it might share the same underlying pathophysiologic mechanism.

In the majority of cases, the patient is typically hospitalized with respiratory failure and coma induced by an overdose of opioids [5,6,9,16]. After supportive treatment, patient enjoys a period of full recovery and is usually discharged home. Brain imaging at this stage fails to find any significant abnormalities with normal white matter signal [8,16]. Days/weeks later (from 12 days to 5 weeks), the patient is then brought with deterioration in neurological status that is manifested mainly by severe cognitive and behavioral changes e.g. odd behavior, personality changes, confusion, memory problems, mutism, hemineglect, catatonia, automatisms, incontinence, abnormal movement and/or gait difficulties [6-10,16]. Those symptoms are associated with the presence of abnormal neurological signs e.g. prominent pyramidal, extrapyramidal and/or cerebellar signs [4-10,13,19]. Occasionally, patient may progress to quadreparesis (typically spastic), stupor and coma [7,9]. Gottfried described a patient who had segmental myoclonus as part of DPHL [7].

Our patient didn’t have lumbar puncture. In patients of DPHL, routine cerebrospinal fluid (CSF) studies (cell count, protein and glucose) are typically normal with negative microbiologic cultures and no evidence of infection including JC virus[10,18], herpes simplex virus (HSV) [5] and Epstein–Barr virus (EBV) [13]. Also, oligoclonal bands are usually absent and CSF IgG levels are within normal [5-8,13]. Shprecher et al. found an evidence of demyelination with elevated CSF myelin basic protein in a patient who had DPHL following opioid-induced coma [13]. EEG classically shows diffuse non-specific slow waves without characteristic findings [5,8,9,13].

The characteristic MRI findings in DPHL include diffuse white matter hyperintensities on T2 sequences involving the periventricular white matter bilaterally which is diffuse and involves bilateral centrum semiovale areas [10,11,20]. Classically, in cases of DPHL, these lesions are restricted to the periventricular subcortical white matter, sparing the cerebellum or the brain stem [4,6,7,13,16,19] and doesn't enhance after contrast administration. On diffusion weighted imaging (DWI), there is a symmetric decrease in the apparent diffusion coefficient (ADC) values in the corona radiata and the centrum semiovale white matter [15]. In addition to areas of restricted water diffusion in both globus pallidi which are usually evident as hypodensities in CT scan [4]. DPHL Cases caused by carbon monoxide poisoning had also the same involvement of periventricular white matter, centrum semiovale and also sparing of the infratentorial structures [2]. This finding might help in differentiating DPHL from the chronic heroin vapor inhalation leukoencephalopathy (chasing-the-dragon toxicity) where there is usually involvement of the infratentorial structures, mainly brainstem and cerebellum [4,21].

Magnetic Resonance Spectroscopy (MRS) in the reported cases of DPHL showed a picture similar to demyelination with an elevated choline peak, increased choline to creatine ratio, elevated lactate and decreased N-acetylaspartate (NAA) [4,7,16]. MRS in heroin vapor (chasing the dragon) toxicity is different, as is showed reduced choline peak, reduced NAA and elevated lactate in previous case reports [4,21].

Pathological studies showed widespread demyelination, with the myelin being replaced by lipid laden macrophages which gives the brain a spongiotic appearance [9]. However, cerebral cortex, putamen, thalamus, hippocampus, brain stem, and cerebellum are usually normal in cases of post-hypoxic leukoencephalopathy [9]. There is no acute of chronic inflammatory changes or white matter necrosis [3,7,9,22]. Occasionally, there is bilateral globus pallidus necrosis that’s is attributed to prolonged hypoxia [3].

The pathophysiology of this phenomenon remains poorly understood. Despite the numerous cases of drug overdose and hypoxic-ischemic brain injury encountered in daily medical practice, this condition remains generally rare. Hence, hypoxia-ischemia alone or opioid overdose alone is not sufficient to explain the unusual selective white matter damage. We believe that this condition will be better explained by a “double hit” mechanism where there is an inherent pre-predisposition leading to a susceptible myelin or myelin-forming cells. This theory was enforced when some patients with DPHL found to be partially deficient of arylsulfatase A, an enzyme which its complete absence causes accumulation of sulfatides in the myelin producing cells and subsequent leukodystrophy (metachromatic leukodystrophy, MLD) [7,23]. This notion was challenged by the finding of other patients with DPHL but with normal arylsulfatase A level [8,10,19]. This leads to other authors suggesting different mechanisms trying to explain the delayed onset of symptoms. Heckmann *et al.* attributed the biphasic course to the slower necrosis of myelin sheath given the longer half-life of myelin that follows the immediate death of myelin-producing glial cells at the border zones of the white matter, which is less perfused compared to gray matter [19,24]. Although the majority of patients with post-hypoxic leukoencephalopathy following drug overdose are of the delayed type, it is unclear why do some patients develop early/acute disease. In the case of our patient, he was brought with severe hypoxia, hypercapnia, acidosis and multiorgan failure which could imply prolonged period of hypoxia and hypoperfusion. This patient is similar to 3 other patients in the literature (see patient #1 [3], patient #2 [18] patient #3 [4]) where the findings appeared earlier in the disease course and there was no recovery to baseline. Our patient and the other three share some features including the unknown duration of coma/hypoxia, late presentation, and evidence of hemodynamic shock and fever. This is in contrast to the majority of cases of delayed presentation DPHL, the hypoxic event (mild-moderate), coma and ICU stay is usually brief (1–3 days) and patients rarely necessitate aggressive resuscitation or inotropic support [4,10]. We assume that in a patient who has some predisposition to leukoencephalopathy (e.g. genetic, use of drugs), a prolonged hypoxia, delayed onset of resuscitation, and/or multiorgan failure might be a risk factor for the early development of leukoencephalopathy. And those patients generally do poorly compared to typical DPHL likely due the presence of an accompanying hypoxic anoxic encephalopathy from the prolonged hypoxia and hypoperfusion.

The role of cannabis in our case is unclear, but we believe it is of a less significance than benzodiazepines for the following reasons; the first being cannabis is known to cause coma only in the children. Secondly, the amount of the THC found in our patient is relatively small and is not typically associated with decrease level of consciousness. For example, Carstairs et al. reported a 14-month-old girl who had decreased level of consciousness (GCS 7) secondary to accidental cannabis ingestion. Her urinary THC-COOH level was 3844 ng/mL and her clinical improvement coincided with a marked decline in the level of urinary THC-COOH to 203 ng/mL [25]. Lastly, the patient was intoxicated with another well-known CNS depressant in a significantly higher quantity which better accounts for the clinical picture. Nevertheless, it is not possible, at this point, to totally eliminate an undescribed role of THC in the pathogenesis of leukoencephalopathy.

Due to the limited number of cases and the poorly understood pathophysiology, no single treatment is shown to be effective and the management remains mainly symptomatic and supportive e.g. antispasticity measures, respiratory and swallowing support. High-dose methylprednisolone [7] and coenzyme Q [4] have been tried with no significant effect.

The majority of DPHL patients in the previous case reports had a course of slow recovery to near baseline. Within weeks, they gradually start to regain some of the functions e.g. speech, ambulate. After 1–2 years, the majority return back to their baseline with some residual cognitive dysfunction (e.g. short-term memory) or fatigue which might render them dependent. Rarely, the patient could have progressive neurological deterioration that eventually lead to death within few weeks from the onset of the delayed phase [9]. The MRI changes usually resolve by this time but with an evidence of diffuse cortical atrophy [5-8,10,13,15].

## Consent

A written informed consent was obtained from the patient’s father for publication of this case report and any accompanying images. A copy of the written consent is available for review by the editor of this journal.

## Abbreviations

DPHL: Delayed post-hypoxic leukoencephalopathy
MRI: Magnetic resonance imaging
DWI: Diffusion weighted imaging
CT: Computerized tomography
ADC: Apparent diffusion coefficient
MRS: Magnetic resonance spectroscopy
NAA: N-acetylaspartate
WBC: White blood cell
pCO2: Partial pressure of carbon dioxide
pO2: Partial pressure of oxygen
ALP: alkaline phosphatase
GGT: Gamma-glutamyl transpeptidase
PT: Prothrombin time
APTT: Activated Partial Thromboplastin Time
CO: Carbon monoxide
ICU: Intensive care unit
CSF: Cerebrospinal fluid
HIV: Human immunodeficiency virus
ELISA: Enzyme-linked immunosorbent assay
EEG: Electroencephalogram
JC virus: John Cunningham virus
HSV: Herpes simplex virus
EBV: Epstein-Barr virus (EBV)
THC: Tetrahydrocannabinol

## References

[1] Sioka C, Kyritsis AP (2009). Central and peripheral nervous system toxicity of common chemotherapeutic agents. Cancer Chemother Pharmacol.

[2] Kim JH, Chang KH, Song IC, Kim KH, Kwon BJ, Kim HC (2003). Delayed encephalopathy of acute carbon monoxide intoxication: diffusivity of cerebral white matter lesions. AJNR Am J Neuroradiol.

[3] Ginsberg MD (1979). Delayed neurological deterioration following hypoxia. Adv Neurol.

[4] Bartlett E, Mikulis DJ (2005). Chasing "chasing the dragon" with MRI: leukoencephalopathy in drug abuse. British J Radiol.

[5] Barnett MH, Miller LA, Reddel SW, Davies L (2001). Reversible delayed leukoencephalopathy following intravenous heroin overdose. J Clin Neurosci Off J Neurosurgical Soc Aust.

[6] Chang WL, Chang YK, Hsu SY, Lin GJ, Chen SC (2009). Reversible delayed leukoencephalopathy after heroin intoxication with hypoxia: a case report. Acta Neurologica Taiwanica.

[7] Gottfried JA, Mayer SA, Shungu DC, Chang Y, Duyn JH (1997). Delayed posthypoxic demyelination. Association with arylsulfatase A deficiency and lactic acidosis on proton MR spectroscopy. Neurology.

[8] Lee HB, Lyketsos CG (2001). Delayed post-hypoxic leukoencephalopathy. Psychosomatics.

[9] Rizzuto N, Morbin M, Ferrari S, Cavallaro T, Sparaco M, Boso G (1997). Delayed spongiform leukoencephalopathy after heroin abuse. Acta Neuropathol.

[10] Wallace IR, Dynan C, Esmonde T (2010). One confused patient, many confused physicians: a case of delayed post-hypoxic leucoencephalopathy. QJM Monthly J Assoc Physicians.

[11] Huang BY, Castillo M (2008). Hypoxic-ischemic brain injury: imaging findings from birth to adulthood. Radiographics A Rev Publ Radiol Soc North Am Inc.

[12] Okuda S, Ueno M, Hayakawa M, Araki M, Kanda F, Takano S (2012). Delayed posthypoxic leukoencephalopathy: case reports. Rinsho Shinkeigaku = Clin Neurol.

[13] Shprecher DR, Flanigan KM, Smith AG, Smith SM, Schenkenberg T, Steffens J (2008). Clinical and diagnostic features of delayed hypoxic leukoencephalopathy. J Neuropsychiatry Clin Neurosci.

[14] Salazar R, Dubow J (2012). Delayed posthypoxic leukoencephalopathy following a morphine overdose. J Clin Neurosci Off J Neurosurgical Soc Aust.

[15] McKinney AM, Kieffer SA, Paylor RT, SantaCruz KS, Kendi A, Lucato L (2009). Acute toxic leukoencephalopathy: potential for reversibility clinically and on MRI with diffusion-weighted and FLAIR imaging. AJR Am J Roentgenol.

[16] Chen-Plotkin AS, Pau KT, Schmahmann JD (2008). Delayed leukoencephalopathy after hypoxic-ischemic injury. Arch Neurol.

[17] Roychowdhury S, Maldjian JA, Galetta SL, Grossman RI (1998). Postanoxic encephalopathy: diffusion MR findings. J Comput Assist Tomogr.

[18] Ryan A, Molloy FM, Farrell MA, Hutchinson M (2005). Fatal toxic leukoencephalopathy: clinical, radiological, and necropsy findings in two patients. J Neurol Neurosurg Psychiatry.

[19] Heckmann JG, Erbguth F, Neundorfer B (1998). Delayed postanoxic demyelination registry. Neurology.

[20] Tormoehlen LM (2011). Toxic leukoencephalopathies. Neurol Clin.

[21] Offiah C, Hall E (2008). Heroin-induced leukoencephalopathy: characterization using MRI, diffusion-weighted imaging, and MR spectroscopy. Clin Radiol.

[22] Nuytten D, Wyffels E, Michiels K, Ferrante M, Verbraeken H, Daelemans R (1998). Drug-induced spongiform leucoencephalopathy, a case report with review of the literature. Acta Neurol Belg.

[23] Weinberger LM, Schmidley JW, Schafer IA, Raghavan S (1994). Delayed postanoxic demyelination and arylsulfatase-A pseudodeficiency. Neurology.

[24] White BC, Grossman LI, Krause GS (1993). Brain injury by global ischemia and reperfusion: a theoretical perspective on membrane damage and repair. Neurology.

[25] Carstairs SD, Fujinaka MK, Keeney GE, Ly BT (2011). Prolonged coma in a child due to hashish ingestion with quantitation of THC metabolites in urine. J Emergency Med.

